# COVID-19 Vaccination Intention in a Community Cohort in Ponce, Puerto Rico

**DOI:** 10.4269/ajtmh.22-0132

**Published:** 2022-07-11

**Authors:** Liliana Sánchez-González, Chelsea G. Major, Dania M. Rodriguez, Abirami Balajee, Kyle R. Ryff, Olga Lorenzi, Mariely Linares, Laura E. Adams, Vanessa Rivera-Amill, Melissa Rolfes, Gabriela Paz-Bailey

**Affiliations:** ^1^ Dengue Branch, Centers for Disease Control and Prevention, San Juan, Puerto Rico;; ^2^ COVID-19 Emergency Response Team, Centers for Disease Control and Prevention, Atlanta, Georgia;; ^3^ Department of Basic Sciences, Ponce Health Sciences University, Ponce, Puerto Rico

## Abstract

As of January 20, 2022, > 247,000 confirmed COVID-19 cases and 3,600 deaths were reported in Puerto Rico (PR). We interviewed participants aged ≥ 14 years in the Communities Organized to Prevent Arboviruses (COPA) study, a community-based cohort in PR, about COVID-19 vaccine intention from November 12, 2020, to June 25, 2021. We used univariate and adjusted analyses to identify participant characteristics associated with vaccine intention. Among 1,542 respondents, the median age was 37 years (interquartile range 23–45) and 914 (59%) were female. Most participants (83%) reported a willingness to receive a COVID-19 vaccine. The most common reason for vaccine hesitancy was concern about the safety or side effects (64%). Willingness to receive the COVID-19 vaccine was associated with a later interview date, higher household income, previous COVID-19 diagnosis among household members, COVID-19 risk perception, influenza vaccine uptake, dengue vaccine intention, and general positive perceptions of vaccines. While parents with minors (< 21 years old) were less likely to report vaccine intention for themselves than participants without minor children, we observed similar characteristics associated with parents’ willingness to vaccinate their children. Overall, COVID-19 vaccine intention was high among COPA participants. It is important that public health messaging in PR addresses COVID-19 vaccine safety and possible side effects.

## INTRODUCTION

As of January 20, 2022, more than 336 million cases of coronavirus disease 2019 (COVID-19), including over 5.5 million associated deaths have been reported globally.^1^ In the United States, more than 68 million cases and 856,000 deaths have been reported, with over 247,000 confirmed cases and 3,631 deaths occurring in Puerto Rico.^2^^,^^3^ Two messenger RNA (mRNA) vaccines against COVID-19 manufactured by Pfizer-BioNTech and Moderna, and one viral vector vaccine manufactured by Janssen/Johnson & Johnson received an Emergency Use Authorization (EUA) from the U.S. Food and Drug Administration (FDA), and the Advisory Committee on Immunization Practices (ACIP) issued interim recommendations for their use in 2020. Following ACIP recommendations for allocating limited vaccines, Puerto Rico started Phase 1A of COVID-19 vaccination of healthcare workers on December 15, 2020, and expanded to other population segments as vaccinations progressed. Currently, all persons 5 years of age and older are eligible to receive a COVID-19 vaccine series and persons 12 years of age and older are eligible for an additional booster dose, according to ACIP guidance.^4^

Vaccine hesitancy, defined as a delay in acceptance or refusal of vaccines despite the availability of vaccines, was identified as one of the 10 most important global health threats by the WHO in 2019.^5^ Surveys conducted in the United States between April and July 2020—before the availability of any COVID-19 vaccine—showed overall vaccine intention for a hypothetical COVID-19 vaccine was between 57% and 79%.^6^^–^^9^ In these studies, between 10% and 16% of respondents were Latino or Hispanic. Later, a nationally representative survey of adults in the United States found that COVID-19 vaccine intention increased overall from September 2020 (39%) to December 2020 (49%), with the largest increase (17%, from 49.1% to 66.2%) among adults over 65 years of age.^10^ Limited studies are available regarding COVID-19 vaccine intention in Latin America before or after the availability of COVID-19 vaccines. One analysis from a social media site’s online survey conducted in early 2021 reported an overall COVID-19 vaccine intention of 80% in Latin America and the Caribbean,^11^ with high vaccine intention prevalence among Puerto Rico’s respondents (85%). Similar vaccine intention prevalence (85.5%) was observed in a small online survey of Puerto Rico residents also conducted in early 2021.^12^

In this study, we aim to determine the level of COVID-19 vaccine intention, the main factors associated with willingness to be vaccinated, and the changes over time among participants in a community cohort implemented to investigate the burden of dengue and other arboviral infections in Ponce, Puerto Rico. As widespread vaccination against COVID-19 is an essential tool to prevent SARS-CoV-2–associated morbidity and mortality and to control the global pandemic, we collected information to help guide public health messaging related to COVID-19 vaccination in Puerto Rico and increase vaccine uptake on the island.

## MATERIALS AND METHODS

Communities Organized to Prevent Arboviruses (COPA) is a community-based prospective cohort study in Ponce, Puerto Rico, that seeks to assess the risk of infection with dengue (caused by any of four dengue virus serotypes mainly transmitted by *Aedes* mosquitoes) and other arboviruses, and provide a platform to evaluate vector control and preventive interventions. In 2018, we began recruitment activities among residents of randomly selected homes in 38 selected cluster areas (groups of communities), including neighborhoods and public housing complexes with single- and multifamily residences.^13^ The cluster areas were selected based on the surveillance data of historical arboviral disease incidence.^14^^–^^17^ Study enrollment is only offered to household members aged 1–50 years, as most incident dengue infections are identified in individuals under 50 years of age. Participation of all eligible household members was not required. Cohort follow-up and replacement activities are conducted annually to maintain approximately 3,800 active participants. As part of these activities, COPA staff collect blood samples and administer standardized questionnaires on demographics, health status and recent illness, arbovirus prevention, and travel and mobility for all participants. In June 2020, a sub-study with weekly respiratory specimen collection for SARS-CoV-2 molecular testing was initiated in 15 of the 38 clusters and extended COPA participation to residents over 50 years of age. Sub-study clusters were selected to maximize the number of participating households with one or more members under 18 or over 50 years (to allow for age-stratified analyses of SARS-CoV-2 infection incidence) and based on logistical priorities, including proximity to other selected clusters and ease of access to residential areas. COVID-19 sub-study participants answer all questionnaires administered to regular COPA participants and are included in this study analysis. This study was reviewed by Ponce Medical School Foundation, Inc. Institutional Review Board. All participants provided written consent.^1^

Questions on individual COVID-19 risk perception and vaccine intention were administered to all participating household members aged 14 years and older. Additionally, participants with minor children (defined as persons under 21 years old in Puerto Rico) were asked about their COVID-19 concerns and vaccine intention for their children. Participants were asked to describe their level of concern for getting sick with COVID-19 as either “very,” “somewhat,” “slightly,” or “not at all.” They were also asked, “if there is an approved vaccine for COVID-19 in Puerto Rico, available for $10 or less, would you get it?” Those who responded that they would not get vaccinated, or were unsure if they would, were asked to specify for what reason(s). Interviewers had a list of reason options to select from, including an option to select and specify “other” responses based on the participants’ answers, but were instructed not to read them. Other COVID-19 or vaccine-related questions addressed COVID-19 diagnosis in themselves or household members, general perceptions of vaccines and vaccine safety, influenza vaccine uptake, and intention to get a free or low-cost dengue vaccine.

We analyzed response data for all participants who completed annual study activities and responded to COVID-19 vaccine intention questions from November 12, 2020, when these questions were implemented for the third year of follow-up activities, through June 25, 2021. Unweighted frequencies were calculated for participant responses on demographics, health and recent acute illness, COVID-19 history and perception, and vaccine perception and intention. Pearson χ^2^ tests with Yates’ continuity correction, Fisher’s exact tests, and nonparametric Kruskal–Wallis tests were used to assess differences in demographic characteristics and responses between participants who were unsure and those who would not get a COVID-19 vaccine. The value of *P* < 0.05 was considered statistically significant.

To assess whether COVID-19 vaccine intention changed over time after significant events, we compared vaccine intention responses during three time periods. The first period (November 12 to December 14, 2020) was before a vaccine with an FDA EUA became available in Puerto Rico. The second period (December 15, 2020, to April 11, 2021) was during a phased rollout period prioritizing the vaccination of high-risk residents. The third period was from April 13, 2021, to June 12, 2021, during which all Puerto Rico residents over 16 years of age were eligible for the Pfizer vaccine.

Log-binomial regression models were used to evaluate the association between COVID-19 vaccine intention as a dependent variable, and time period, attitudes, and beliefs about COVID-19, COVID-19 vaccination, and vaccination in general, as independent variables. A full model was estimated including all variables and the backward variable selection was performed to retain variables that would best improve the model. To avoid overadjustment, only significant participant demographics (i.e., sex and income) were included in the final adjusted regression model. Due to limited cell sizes for several categories, particularly for participants unsure or unwilling to vaccinate against COVID-19, some adjusted models failed to converge. Where possible, we reported both univariate relative risks (uRRs) and adjusted relative risks (aRRs) for participants who would versus those who were unsure if they would or would not get the COVID-19 vaccine to identify factors associated with COVID-19 vaccine intention.

All analyses were conducted using R (version 4.0.4)^18^; we used the logbin package to fit the log-binomial regression model, which includes an Expectation Maximization-type algorithm to stabilize convergence properties.^19^

## RESULTS

A total of 1,694 participants completed annual study activities from November 12, 2020, through June 25, 2021; of these, 1,542 (91%) participants from 913 households answered the COVID-19 vaccine intention questions and were included in the analyses. Of the 1,542 COPA participants with available data, 239 (15%) were also part of the COVID-19 sub-study. The median age was 37 years (interquartile range 23–45), 20% (*N* = 300) were minors and 59% (*N* = 914) were female (Table 1). A total of eight participants reported being pregnant (0.5%). Almost all participants (99%) identified as Latino and most (96%) reported having medical insurance. Among the insured, a majority (68%) reported having public medical insurance. Most participants reported being employed (47%), students (18%), or homemakers (16%), and 58% reported an annual household income less than US $20,000 (Table 1).

**Table 1:** Characteristics of COPA participants responding to COVID-19 vaccine intention questions, Ponce, Puerto Rico, December 2020 to June 2021 (*N* = 1,542)

	No. participants	Percentage (%)*	Puerto Rico Population (%)†
Sex			
Female	914	59.3	52.5
Male	628	40.7	–
Age (*N* = 1,542)			
Median years, interquartile range	37	23–45	–
14–20 years	300	19.5	8.8
21–30 years	284	18.4	13.6
31–40 years	343	22.2	11.6
41–50 years	499	32.3	12.6
51+ years	116	7.5	40
Annual household income (*N* = 1,406)			
< $10,000	538	38.3	Median annual household income: $20,539
$10,000–$19,999	280	19.9	
$20,000–$29,999	264	18.8	
$30,000–$49,999	197	14.0	
$50,000+	127	9.0	
Race (*N* = 1,422)			
White only	912	64.1	65.9
Black only	187	13.2	11.7
Mixed	124	8.7	5.3
Other	68	4.8	0.4%
Unsure	131	9.2	–
Ethnicity (*N* = 1,436)			
Latino	1,419	98.8	98.7
Other	17	1.2	1.3
Education (*N* = 1,491)			
≤ 12th grade or GED	757	50.8	For people over 25 years old—Bachelors or higher: 25.9%
College/technical school	633	42.5	
Postgraduate study/professional degree	101	6.8	
Employment (*N* = 1,494)			
Used‡	705	47.2	44.4
Student	271	18.1	–
Homemaker/caretaker for family	244	16.3	–
Unemployed	208	13.9	–
Other	66	4.4	–
Medical insurance (*N* = 1,493)			
Yes	1,432	95.9	–
No	61	4.1	9.6% (for people < 65 years old)
Type of medical insurance (*N* = 1,429)			
Private insurance	460	32.2	–
Public insurance	969	67.8	–
Reported at least one chronic condition (*N* = 1,468)			
Yes	625	42.6	–
No	843	57.4	–
Self or household member diagnosed with COVID-19 in the past year (*N* = 1,542)			
Yes	85	5.5	–
No	1,457	94.5	–
Had contact with anyone diagnosed with COVID-19 in the last 7 days (*N* = 1,445)			
Yes	9	0.6	–
No	1,436	99.4	–
Had contact with anyone with fever or cough in the last 14 days (*N* = 1,469)			
Yes	36	2.5	–
No	1,433	97.5	–
Risk perception of COVID-19 disease			
*About themselves* (*N* = 1,540)			
Very worried	1,397	90.7	–
Somewhat worried	85	5.5	–
Slightly worried	44	2.9	–
Not worried	14	0.9	–
*About their children* (*N* = 744)			
Very worried	726	97.6	–
Somewhat worried	10	1.3	–
Slightly worried	6	0.8	–
Not worried	2	0.3	–
Would get an approved COVID-19 vaccine if free or low cost			
*For themselves (N = 1,542)*			
Yes	1,274	82.6	–
Unsure	111	7.2	–
No	157	10.2	–
*For their children (N = 740)*			
Yes	593	80.1	–
Unsure	63	8.5	–
No	84	11.4	–

COPA = Communities Organized to Prevent Arboviruses.

* Nonresponses are excluded from the denominator in percentage calculations.

† As reported in https://www.census.gov/quickfacts/PR.

‡ Includes participants who were students and employed.

Regarding COVID-19 risk perception, most participants (91%) reported being very worried about getting COVID-19 disease, and among participants with children under 21 years old (*N* = 744), almost all (98%) reported being very worried about their children getting COVID-19 disease (Table 1). A total of 85 (5%) participants reported that they or a household member were diagnosed with COVID-19 in the last year, and 9 (< 1%) reported having contact with someone diagnosed with COVID-19 in the 7 days prior to the COPA study visit. Among all respondents, 1,274 (82%) reported they would get a COVID-19 vaccine, 157 (10%) said they would not receive a COVID-19 vaccine, and 111 (7%) were unsure (Table 1). Among parents of children under 21 years of age who answered the vaccine intention question about their children (*N* = 740), 593 (80%) reported that they would vaccinate their children, 84 (11%) reported that they would not vaccinate their children, and 63 (8%) reported that they were not sure.

**Figure 1.**
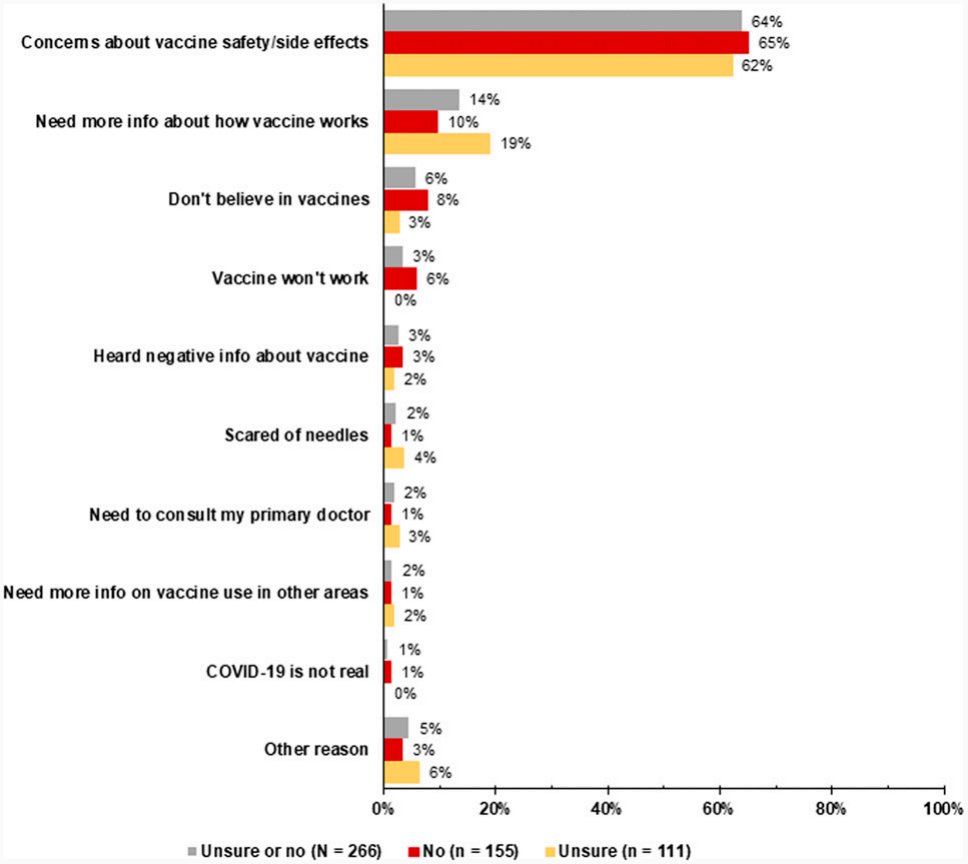
Reasons Communities Organized to Prevent Arboviruses (COPA) participants would not or were unsure if they would get an authorized COVID-19 vaccine that was free or low-cost ($10 or less), November 2020 to June 2021 (*N* = 266)*. *Excludes two participants who did not provide a reason for not getting the COVID-19 vaccine. Participants could give more than one reason. This figure appears in color at www.ajtmh.org.

Overall, the most common reasons for COVID-19 vaccine hesitancy were concerns about safety or side effects (64%), needing more information on how the COVID-19 vaccine works (14%), and not believing in vaccines (6%) (Figure 1). Participants who were unsure about getting a COVID-19 vaccine were more likely to say they needed more information about how the vaccine works than those who would not get the vaccine (19% versus 10%, *P* = 0.03). Participants who would not get the COVID-19 vaccine were more likely to say that they did not believe that the vaccine works than those who were unsure about getting vaccinated (6% versus 0%, *P* = 0.03). Participants who were unsure about getting the COVID-19 vaccine were also more likely to say that they believed FDA-approved vaccines are safe (74% versus 62%, *P* = 0.05), reported intention to get a dengue vaccine (56% versus 35%, *P* = 0.001), and had an annual household income < $10,000 (55% versus 40%, *P* = 0.02) than those who would not get the COVID-19 vaccine. There were no other significant differences regarding reasons for hesitancy or vaccine refusal among participants who were unsure about getting and those who would not get the COVID-19 vaccine.

Univariate analyses of participants who reported that they would get a COVID-19 vaccine (*N* = 1,274) with those who were unsure or would not (*N* = 268), showed that there were significant differences by sex, household income, insurance type, and parental status, and differences by household income and parental status persisted in adjusted analyses (Table 2). Male participants were more likely to report vaccine intention than female participants (85% versus 81%, uRR 0.95; 95% CI 0.91–0.99). Overall, vaccine intention increased with increasing annual household income, with participants reporting an income between $30,000 and $49,999 (88% versus 78% uRR 1.13; 95% CI 1.05–1.20) and > $50,000 (86% versus 78%, uRR 1.09; 95% CI 1.01–1.19) significantly more likely to report vaccine intention compared with those with income ≤ $10,000. After adjusting for sex, a significant difference was only observed among participants reporting an income between $30,000 and $49,999 (aRR 1.12; 95% CI 1.05–1.20). Participants who reported having private insurance were more likely to report vaccine intention than public insurance holders (86% versus 80%, uRR 1.07, 95% CI 1.01–1.12), but no significant difference was observed after adjusting for sex and income. In univariate and adjusted analyses, participants with children under 21 years old were less likely to report vaccine intention for themselves than those without children (80% versus 85%, aRR 0.94, 95% CI 0.89–0.99).

**Table 2:** COVID-19 vaccine intention by demographics and other factors among COPA participants, Ponce, Puerto Rico, December 2020 to June 2021 (*N* = 1,542)

	Participants who would receive COVID-19 vaccine (*N* = 1,274)	Participants who would not or unsure if would receive COVID-19 vaccine (*N* = 268)	Prevalence of COVID-19 vaccine intention	Univariate relative risk (95% CI)	Adjusted relative risk (95% CI) by annual household income and/or sex*
Sex					
Female	739	175	80.9%	0.95 (0.91–0.99)†	1.00 (0.95–1.05)
Male	535	93	85.2%	Reference	Reference
Age group					
14–20 years	252	48	84.0%	Reference	
21–30 years	238	46	83.8%	1.00 (0.93–1.07)	
31–40 years	266	77	77.6%	0.92 (0.86–1.00)	
41–50 years	419	80	84.0%	1.00 (0.94–1.06)	
51+ years	99	17	85.3%	1.02 (0.93–1.11)	
Parents of children < 21 years‡					
Yes	597	149	80.0%	0.93 (0.89–0.98)†	0.94 (0.89–0.99)†
No	424	70	85.8%	Reference	Reference
Annual household income					
< $10,000	422	116	78.4%	Reference	Reference
$10,000–$19,999	230	50	82.1%	1.10 (1.08–1.12)	1.04 (0.97–1.12)
$20,000–$29,999	222	42	84.1%	1.07 (1.00–1.15)	1.07 (1.00–1.15)
$30,000–$49,999	174	23	88.3%	1.13 (1.05–1.20)†	1.12 (1.05–1.20)†
$50,000+	109	18	85.8%	1.09 (1.01–1.19)†	1.09 (1.00–1.18)
Race					
White only	748	164	82.0%	Reference	
Black only	155	32	82.9%	1.01 (0.94–1.09)	
Mixed/Other	163	37	81.5%	0.99 (0.90–1.07)	
Unsure	116	15	88.5	1.08 (1.01–1.16)	
Education					
≤ 12th grade or GED	621	143	81.4%	Reference	Reference
College/technical school or higher degree	613	121	83.5%	1.03 (0.98–1.08)	1.00 (0.95–1.05)
Currently used					
Working§	594	111	84.3%	1.04 (1.00–1.09)	1.01 (0.96–1.06)
Not working	637	152	80.7%	Reference	Reference
Medical insurance					
Yes	1,177	255	82.2%	0.95 (0.86–1.05)	1.00 (0.89–1.12)
No	53	8	86.9%	Reference	Reference
Type of medical insurance					
Private insurance	396	64	86.1%	1.07 (1.01–1.12)†	1.01 (0.95–1.08)
Public insurance	779	190	80.4%	Reference	Reference
Reported at least one chronic condition					
Yes	503	122	80.5%	0.96 (0.92–1.01)	1.00 (0.95–1.05)
No	706	137	83.7%	Reference	Reference
Self or household member diagnosed with COVID-19 in past year					
Yes	77	8	90.6%	1.10 (1.03–1.19)†	
No or unsure	1,197	260	82.2%	Reference	
Had contact with anyone with fever or cough in last 14 days					
Yes	34	2	94.4%	1.15 (1.06–1.25)†	
No or unsure	1,175	258	82.0%	Reference	
Spends 5 or more hours per week at work or other single location outside home					
Yes	527	96	84.6%	1.04 (0.99–1.09)	1.02 (0.97–1.07)
No	673	156	81.2%	Reference	Reference
Risk perception of COVID-19 disease					
Very or somewhat worried	1,232	250	83.1%	1.21 (1.01–1.43)†	1.24 (1.03–1.50)†
Slightly or not at all worried	40	18	69.0%	Reference	Reference
Believe that vaccines are important to prevent certain diseases					
Yes	1,244	221	84.9%	2.25 (1.66–3.05)†	2.20 (1.62–2.99)†
No or unsure	26	43	37.7%	Reference	Reference
Believe that FDA-approved vaccines are safe					
Yes	1,154	176	86.8%	1.51 (1.34–1.70)†	1.50 (1.32–1.70)†
No or unsure	115	85	57.5%	Reference	Reference
Vaccinated against influenza in past year					
Yes	440	56	88.7%	1.12 (1.07–1.17)†	1.12 (1.07–1.17)†
No or unsure	771	203	79.2%	Reference	Reference
Would get free or low-cost dengue vaccine					
Yes	1,164	115	91.0%	2.18 (1.88–2.52)†	2.17 (1.87–5.29)†
No or unsure	107	149	41.8%	Reference	Reference
Time period of interview					
November 12 to December 14, 2020	146	59	71.2%	Reference	Reference
December 15, 2020 to April 11, 2021	650	150	81.3%	1.14 (1.04–1.25)†	1.16 (1.05–1.28)†
April 12 to June 25, 2021	478	59	89.0%	1.25 (1.14–1.37)†	1.25 (1.13–1.38)†

COPA = Communities Organized to Prevent Arboviruses; FDA = Food and Drug Administration.

* Empty cells in the Adjusted Relative Risk column are presented if the model failed to converge for small cell sizes in several categories.

† Relative risk is statistically significant.

‡ Includes adult respondents (21 years and older) only as no participants under this age reported having children.

§ Includes participants that were students and used.

Participants who reported that they or a household member had been diagnosed with COVID-19 in the past year were more likely to report COVID-19 vaccine intention than those who did not in unadjusted analyses (91% versus 82%, uRR 1.10, 95% CI 1.03–1.19); an aRR could not be calculated due to small cell sizes. COVID-19 vaccine intention was also higher among participants who reported being very or somewhat worried about getting sick than among participants who reported being only slightly or not at all worried about getting sick with COVID-19 in univariate and adjusted analyses (83% versus 69%, aRR 1.24, 95% CI 1.03–1.50). No significant differences in COVID-19 vaccine intention were found by education level, employment status, race, ethnicity, or history of chronic conditions.

Participant responses related to general vaccine acceptability and interview timing were significantly associated with COVID-19 vaccine intention before and after adjusting for household income and sex. Participants who answered that vaccines are important to prevent diseases (85% versus 38%, aRR 2.20, 95% CI 1.62–2.99) and that FDA-approved vaccines are safe (87% versus 58%, aRR 1.50, 95% CI 1.32–1.70) were also more likely to report vaccine intention when compared with those who answered they were not important or safe. Participants who reported getting an influenza vaccine in the last year were more likely to report the intention to get a COVID-19 vaccine compared with those who did not (89% versus 79%, aRR 1.12, 95% CI 1.07–1.17), as were those who reported they would get a dengue vaccine when available compared with those who would not (91% versus 42%, aRR 2.17, 95% CI 1.87–5.29). Participants who were interviewed from December 15, 2020, to April 11, 2021, when COVID-19 vaccine rollout began in Puerto Rico (81% versus 71%, aRR 1.16, 95% CI 1.05–1.28), and from April 12 to June 25, 2021, when the COVID-19 vaccine became available to all age-eligible residents (89% versus 71%, aRR 1.25, 95% 1.13–1.38), were more likely to report COVID-19 vaccine intention than those interviewed before the vaccine became available.

Most parents who reported that they would be vaccinated against COVID-19 also reported that they would vaccinate their children (96%) (Table 3). In univariate analyses, parents’ intention to vaccinate their children increased with parents’ increasing age, with parents 41–50 and ≥ 51 years old more likely to report vaccine intention for their children than those 21–30 years old (85% and 91% versus 72%, respectively, uRR 1.18; 95% CI 1.04–1.34 and uRR 1.27, 95% CI 1.09–1.48); aRRs could not be calculated to small cell sizes. There were no significant differences by annual household income in parents’ intent to vaccinate their children against COVID-19.

**Table 3:** Intention to vaccinate children against COVID-19 by demographics and other factors among COPA adult participants with children under 21 years, Ponce, Puerto Rico, December 2020 to June 2021 (*N* = 744)

	Participants who would vaccinate children against COVID-19 (*N* = 594)	Participants who would not or unsure if would vaccinate children against COVID-19 (*N* = 150)	Prevalence of COVID-19 vaccine intention	Univariate relative risk (95% CI)	Adjusted relative risk (95% CI) by annual household income and/or sex*
Sex					
Female	388	112	77.6%	0.92 (0.86–0.99)†	1.00 (0.93–1.07)
Male	206	38	84.4%	Reference	Reference
Age group					
21–30 years	76	30	71.7%	Reference	
31–40 years	205	66	75.6%	1.06 (0.92–1.21)	
41–50 years	273	50	84.5%	1.18 (1.04–1.34)*	
51+ years	40	4	90.9%	1.27 (1.09–1.48)†	
Annual household income					
< $10,000	201	56	78.2%	Reference	Reference
$10,000–$19,999	106	36	74.6%	0.95 (0.85–1.07)	0.95 (0.84–1.06)
$20,000–$29,999	105	23	82.0%	1.05 (0.95–1.16)	1.05 (0.95–1.16)
$30,000 to $49,999	91	17	84.3%	1.08 (0.97–1.20)	1.06 (0.96–1.18)
$50,000+	63	10	86.3%	1.10 (0.99–1.23)	1.08 (0.97–1.21)
Education					
≤ 12th grade or GED	206	61	77.2%	Reference	Reference
College/technical school or higher degree	370	86	81.1%	1.05 (0.97–1.14)	1.02 (0.94–1.12)
Self or household member diagnosed with COVID-19 in the past year					
Yes	44	5	89.8%	1.13 (1.02–1.26)†	1.13 (1.06–1.21)†
No or unsure	550	145	79.1%	Reference	Reference
Had contact with anyone with fever or cough in the last 14 days					
Yes	17	2	89.5%	1.13 (0.96–1.32)	
No or unsure	550	144	79.3%	Reference	
Spends 5 or more hours per week at work or other location outside home					
Yes	284	67	80.9%	1.03 (0.96–1.11)	1.01 (0.94–1.09)
No	282	78	78.3%	Reference	Reference
Risk perception of COVID-19 disease for their children					
Very or somewhat worried	590	146	80.2%	1.60 (0.80–3.21)	1.62 (0.81–3.27)
Slightly or not at all worried	4	4	50.0%	Reference	Reference
Believe that vaccines are important to prevent certain diseases					
Yes	575	128	81.8%	1.91 (1.24–2.93)†	1.99 (1.36–2.93)†
No or unsure	12	16	42.9%	Reference	Reference
Believe that FDA-approved vaccines are safe					
Yes	528	108	83.0%	1.36 (1.16–1.59)†	1.34 (1.15–1.56)†
No or unsure	63	40	61.2%	Reference	Reference
Vaccinated against influenza in past year					
Yes	198	31	86.5%	1.13 (1.05–1.22)†	1.12 (1.04–1.20)†
No or unsure	371	115	76.3%	Reference	Reference
Would vaccinate child against dengue					
Yes	552	65	89.5%	2.75 (2.13–3.55)†	2.64 (2.05–3.40)†
No or unsure	40	83	32.5%	Reference	Reference
Children’s vaccines schedule up to date					
Yes	566	137	80.5%	1.18 (0.95–1.46)	1.20 (0.97–1.48)
No or unsure	28	13	68.3%	Reference	Reference
Would vaccinate self against COVID-19					
Yes	570	21	96.4%	6.25 (4.26–9.10)	
No or unsure	23	126	15.4%	Reference	
Time period of interview					
November 12 to December 14, 2020	71	29	71.0%	Reference	Reference
December 15, 2020 to April 11, 2021	288	82	77.8%	1.10 (0.96–1.26)	1.07 (0.94–1.23)
April 12 to June 25, 2021	234	36	86.7%	1.22 (1.07–1.40)†	1.17 (1.02–1.34)†

COPA = Communities Organized to Prevent Arboviruses; FDA = Food and Drug Administration.

* Empty cells in the Adjusted Relative Risk column are presented if the model failed to converge for small cell sizes in several categories.

† Relative risk is statistically significant.

In univariate and adjusted analyses, parents of children under 21 years who reported that they or a household member were diagnosed with COVID-19 in the past year were more likely to report COVID-19 vaccine intention for their children than those who did not (90% versus 80%, aRR 1.13, 95% CI 1.06–1.21) (Table 3). As observed from participants reporting COVID-19 vaccine intention for themselves, parental intention to vaccinate their children against COVID-19 was also consistently positively associated with perceived importance and trust of vaccines generally. Parents who reported intention to vaccinate their children against dengue if an approved free or low-cost dengue vaccine was available were also more likely to report intention to vaccinate their children against COVID-19 than those who would not or were unsure if they would vaccinate their children against dengue (90% versus 33%, aRR 2.64, 95% CI 2.05–3.40). Most parents reported that their children’s vaccines were up to date (80%). There were no observed associations between participants’ responses to this question and whether they would vaccinate their children against COVID-19. Compared with participants interviewed before the COVID-19 vaccine was available in Puerto Rico, vaccine intention for their children was significantly higher among participants interviewed after April 11, when the COVID-19 vaccine became available for all age-eligible residents in Puerto Rico (87% versus 71%, aRR 1.17, 95% CI 1.02–1.34), but not among participants interviewed from December 15, 2020, and April 11 before and after adjusting for sex and household income.

## DISCUSSION

We found a high level of COVID-19 vaccine intention, both for participants and for their children, in a community cohort in Ponce, Puerto Rico. We also found a corresponding high level of concern about getting sick with COVID-19. In our study, participants’ intention to vaccinate themselves against COVID-19 was associated with income and interview timing as the COVID-19 vaccine became increasingly available in Puerto Rico. Other factors positively associated with participants’ intention to vaccinate themselves against COVID-19 include a history of COVID-19 illness in a household member, general positive perceptions of vaccines, influenza vaccine uptake, and willingness to receive a dengue vaccine. Overall, parents of children under 21 years were less likely to report vaccine intention for themselves than nonparents, but similar factors were associated with parents’ intention to vaccinate their children against COVID-19 as for participants’ intention to vaccinate themselves. The most common reason for COVID-19 vaccine hesitancy in this population was concern related to vaccine safety and side effects.

Overall, 82% of our participants reported COVID-19 vaccine intention, higher than what has been previously reported overall in the United States (50%),^10^^,^^20^ with intention increasing significantly after the availability of vaccines in Puerto Rico in mid-December 2020. These findings align with a recent analysis of online surveys in Latin America which found that among 7,000 Puerto Rican respondents, COVID-19 vaccine intention was 85%, slightly higher than the average in the region (80%),^11^ and with a report that found a vaccine intention among Puerto Ricans was 68% before vaccine availability.^21^ We also found that most parents (96%) who would vaccinate themselves were also willing to vaccinate their children, which is consistent with a recent multistate survey found that parents’ willingness to vaccinate their children closely matched their vaccine intention.^22^

Determinants of vaccine intention include individual, context-level, and vaccine-specific aspects^23^ and vary across geographical regions. Similar to other reports from the United States and other countries, participants reporting higher income in our study were more likely to be willing to receive a COVID-19 vaccine.^20,21,24–26^ While some authors have suggested that higher COVID-19 vaccine intention among higher income individuals may be due to higher mobility and the perception that receiving the vaccine less costly than social distancing measures,^27^ neither employment status nor spending 5 or more hours per week at a location outside of the home was associated with COVID-19 vaccine intention in our study population. Maintaining policies to ensure that COVID-19 vaccines are free of any fee or ancillary cost and increasing convenience and availability by bringing vaccination campaigns to neighborhoods and/or providing services like free transportation could increase vaccine uptake in low-income populations. Cost-free vaccination is likely crucial in the context of Puerto Rico, where 43% of the population lives below the poverty line and the estimated median annual household income is $20,539, the lowest of any state or territory in the United States.^28^ Additionally, participants who were unsure about being vaccinated more frequently reported a low annual household income (< $10,000) than those who said they would not get vaccinated. These participants were also more likely to give other responses suggesting that they may be more reachable for COVID-19 vaccine efforts than those who said they would not be vaccinated, including a need for more information on how the COVID-19 vaccine works. These results further support the need for educational campaigns to reach low-income populations.

Similar to our findings, it has been reported previously that parents are more likely to be hesitant about vaccines, including COVID-19 vaccines compared with nonparents, particularly parents with younger children.^22^ Thus, the lower COVID-19 vaccine intention for children in the younger parent age groups we observed may be related to younger child age, and generational differences in vaccine perceptions and exposure to anti-vaccine materials. The lack of available safety data for available vaccines among children under 12 years old during the interview period may also contribute to this finding.

We found that participants were more willing to get vaccinated if someone in the household had been diagnosed with COVID-19. Previous global and U.S. surveys conducted before the availability of COVID-19 vaccines did not find an association between previous COVID-19 diagnosis and vaccine intention,^24^^,^^29^ and others have excluded people with previous COVID-19 diagnosis from vaccine intention studies because of concerns regarding confusion of intention versus need for a vaccine.^30^ The association we found may be related to increased risk perception and aversion to the disease after a personal experience. An educational campaign by the Puerto Rico Department of Health among providers and the general public that included the need to vaccinate against COVID-19 even among those with a history of the disease may also have impacted these findings.

As expected, participants with positive perceptions toward vaccines, including believing in their importance in preventing disease and their safety, once authorized by the FDA, reported higher vaccine intention in our study. Several studies worldwide have found similar associations between positive attitudes toward vaccines, including attitudes toward safety, and higher acceptance of vaccines, including the COVID-19 vaccine.^7,8,26,31^

The association between influenza vaccine uptake and COVID-19 vaccine intention in our study has been shown in other studies in the United States^7^^,^^32^ and may be related to higher risk perception for respiratory diseases among people who vaccinate against influenza, and general vaccine confidence. Similarly, we found a significant association between COVID-19 vaccine intention and participants’ willingness to vaccinate themselves and their children against dengue.

COVID-19 vaccination is ongoing and all residents of Puerto Rico who are 5 years and older are eligible to be vaccinated. As of January 11, 2022, more than 2,400,000 people have received a completed COVID-19 vaccine series,^3^ which corresponds to about 81% of the eligible population in the island.^33^ As COVID-19 vaccine availability is widespread, and additional “booster” doses are recommended to maintain vaccine-induced immunity, there is a continued need for information on reasons for unwillingness to vaccinate to inform public health strategies for increasing vaccine coverage. The main reasons for COVID-19 vaccine hesitancy reported by our participants were concerns about safety and side effects, and the need for more information about how vaccines work. This finding is aligned with the increase in vaccine intention we observed after the availability of the first COVID-19 vaccine in Puerto Rico, when more information on vaccine safety and effectiveness became publicly available. This also aligns with previous reports where safety concerns continue to be the main reason for vaccine reluctance or deliberation.^7,10,32,34^ These results emphasize the need for clear, simple, and accurate information on vaccine safety and side effects, and can be useful when designing and implementing educational and informational campaigns to increase vaccine uptake in the Puerto Rican population.

This study is subject to some limitations. Due to the nature of the COPA study, participants generally have repeated contact with study personnel and their answers may be subject to social desirability bias. However, participants are assured before the interviews that there are no “wrong” or “right” answers and are encouraged to answer honestly. Moreover, our cohort’s reported level of vaccine intention is similar to what has been reported in Latin America generally. Therefore, we do not expect this bias to have had a significant impact on our results. We did not adjust relative risk results to account for household-level clustering in responses and characteristics, which may have led to the overrepresentation of participants with certain household-level characteristics, such as income, in one of the vaccine intention groups. Additionally, our surveyed population is not a representative sample of the Ponce or Puerto Rico population, and younger age groups were overrepresented due to the original study design. Nonetheless, with new age groups now eligible to be vaccinated and the need for “booster” doses, information on factors associated with the unwillingness to receive a COVID-19 vaccine at the jurisdiction level can be helpful to redirect and create new strategies that increase vaccine uptake and reach target vaccination levels, especially in areas with a high burden of disease.

In conclusion, COVID-19 disease concern and vaccine intention were high among participants of our cohort in Ponce, Puerto Rico. As high vaccine coverage is necessary to control the current COVID-19 pandemic, factors associated with vaccine intention found in this study can be used by public health authorities in Puerto Rico to design and tailor messages and strategies to increase vaccine uptake. These strategies are particularly needed among lower-income and publicly insured populations to decrease inequalities in vaccination coverage. Messages can include specific information on vaccine safety and side effects, which was the primary concern for persons in this population who were hesitant to receive a COVID-19 vaccine.
